# Characterization of cytotoxic *Citrobacter braakii* isolated from human stomach

**DOI:** 10.1002/2211-5463.13770

**Published:** 2024-01-24

**Authors:** Mengchao Yu, Fangyu Xie, Chengzhen Xu, Ting Yu, Yixuan Wang, Shuzhen Liang, Quanjiang Dong, Lili Wang

**Affiliations:** ^1^ Central Laboratories and Department of Gastroenterology, Qingdao Municipal Hospital University of Health and Rehabilitation Sciences Qingdao China; ^2^ Department of Cardiology, Qingdao Municipal Hospital University of Health and Rehabilitation Sciences Qingdao China; ^3^ Department of Chinese Medicine Qingdao No. 6 People's Hospital China

**Keywords:** adhesion, *Citrobacter braakii*, cytotoxicity, genome, interleukin‐8

## Abstract

*Citrobacter braakii* (*C. braakii*) is an anaerobic, gram‐negative bacterium that has been isolated from the environment, food, and humans. Infection by *C. braakii* has been associated with acute mucosal inflammation in the intestine, respiratory tract, and urinary tract. However, the pathogenesis of *C. braakii* in the gastric mucosa has not yet been clarified. In this study, the bacterium was detected in 35.5% (61/172) of patients with chronic gastritis (CG) and was closely associated with the severity of mucosal inflammation. *Citrobacter braakii* P1 isolated from a patient with CG exhibited urease activity and acid resistance. It contained multiple secretion systems, including a complete type I secretion system (T1SS), T5aSS and T6SS. We then predicted the potential pilus‐related adhesins. *Citrobacter braakii* P1 diffusely adhered to AGS cells and significantly increased lactate dehydrogenase (LDH) release; the adhesion rate and LDH release were much lower in HEp‐2 cells. Strain P1 also induced markedly increased mRNA and protein expression of *IL‐8* and *TNF‐α* in AGS cells, and the fold increase was much higher than that in HEp‐2 cells. Our results demonstrate proinflammatory and cytotoxic role of *C. braakii* in gastric epithelial cells, indicating the bacterium is potentially involved in inducing gastric mucosa inflammation.

## Graphical abstract

*Citrobacter braakii* is prevalent in the stomach and is associated with the severity of gastric mucosal inflammation. *Citrobacter braakii* adheres to gastric epithelial cells and induces the release of the lactate dehydrogenase and proinflammatory cytokines IL‐8 and TNF‐α, indicating the bacterium is potentially involved in the occurrence of gastric mucosa inflammation.

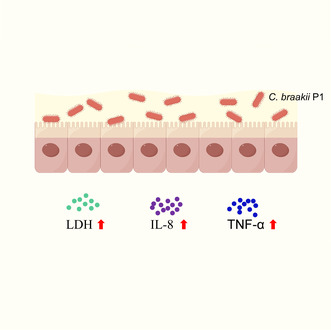

**Abbreviations**
CDS: coding sequence
CG: chronic gastritis
LB: Luria–Bertani
LDH: lactate dehydrogenase
MOI: multiplicity of infection
OR: odds ratio
PBS: phosphate‐buffered saline
T1SS: type I secretion system
TEM: transmission electron microscopy
UBT: urea breath test

The human microbiome plays vital roles in health and disease. In the stomach, the mucosa‐associated microbiome contains hundreds of bacterial species [1, 2]. Disruption of the gastric microbiome is closely associated with the development of mucosal inflammation and carcinogenesis [3, 4, 5]. Oral bacteria, urease‐containing bacteria, and nitrate‐reducing bacteria have been shown to be overrepresented in the dysbiotic microbiome of stomach and gut [6, 7, 8]. Persistent ureases catalyze the conversion of urea to ammonia and carbon oxide, facilitating bacterial survival and proliferation in the acidic stomach [9, 10, 11]. Despite a close association between urease‐containing bacteria and gastric diseases, few studies have been conducted to explore the pathogenesis of urease‐containing bacteria in the gastric epithelium.

The persistence of *Citrobacter* species in the microbiome has been observed in the stomach, intestine, oral cavity, and respiratory tract [12, 13, 14, 15]. Analyses of the gastric microbiome have revealed that *Citrobacter* is predominant in *Helicobacter pylori* (*H. pylori*)‐negative patients with chronic superficial gastritis [16]. Laura Conti *et al*. [17] compared the composition of the gastric microbiome between 23 patients with atrophic gastritis and 32 patients with nonatrophic gastritis. Only one patient from each group was positive for *H. pylori*. The results showed enrichment of *Citrobacter* in patients with atrophic gastritis and a positive correlation with the degree of gastric mucosal atrophy and intestinal metaplasia, indicating a pathogenic role of bacterial persistence in the development of precancerous lesions. Ferreira *et al*. [18] previously demonstrated that *Citrobacter* is the dominant genus in patients with gastric cancer, exhibiting a marked increase in relative abundance compared with chronic gastritis (CG). The adjusted odds ratio (OR) value for the presence of *Citrobacter* in patients with stomach cancer was 6.5, indicating a strong correlation between the overgrowth of *Citrobacter* and gastric cancer.

*Citrobacter braakii* (*C. braakii*) is an anaerobic, gram‐negative bacterium that has been isolated from the environment, food, and humans [19]. Genomic analyses have shown that the bacterium contains a urease cluster. Infection by *C. braakii* has been associated with acute mucosal inflammation in the intestine, respiratory tract, and urinary tract [20, 21, 22]. In immunocompromised patients, bacterial colonization causes bacteremia and inflammation of transplanted organs [23, 24]. Certain isolates have exhibited cytotoxic effects and the ability to adhere to HEp‐2 cells [21, 22, 25]. However, the pathogenesis of *C. braakii* in the gastric mucosa has not yet been clarified. The aim of this study was to characterize the pathogenic potential of *C. braakii* in the gastric epithelium.

## Materials and methods

### Patients and sample

A total of 172 patients with CG were enrolled in the study. These patients underwent endoscopic examination at Qingdao Municipal Hospital due to complaints of upper gastrointestinal symptoms. Of these patients, 91 were male, and the mean age was 50.4 ± 13.8 years. Histological examinations were used to determine the *H. pylori* status. A total of 58 patients were positive for *H. pylori*, and 114 were negative for *H. pylori*. None of these patients had received any antibiotics or proton pump inhibitor treatment 8 weeks prior to the examination. The endoscopy findings for CG patients showed no local lesions (ulcer, polyp, or bleeding). During endoscopic examination, two antral biopsy samples were taken. One sample was stored at −80 °C for genomic DNA extraction until use. The other biopsy sample was used for histological examination. The severity or activity of gastric mucosal inflammation was scored from 0 to 3 according to the number of mononuclear cells or neutrophils infiltrating the lamina propria, respectively [26].

### Ethics statement

The study was conducted in accordance with The Code of Ethics of the World Medical Association (Declaration of Helsinki). All patients written informed consent to take gastric antral biopsy specimens during diagnostic upper gastrointestinal endoscopy. The Ethics Committee of Qingdao Municipal Hospital issued ethical approvals (permit number 2022‐002).

### Detection of *C. braakii* in gastric mucosa

Genomic DNA was extracted from an antral biopsy sample using a Qiagen DNeasy Blood and Tissue Kit (Qiagen, Hilden, Germany; Cat#69504). To eliminate potential DNA contamination, 1 U DNase I was used to treat ground biopsy samples. Prior to detecting *C. braakii* in the gastric mucosa, a PCR method was developed. A pair of primers specifically targeting *C. braakii cfa* was designed using Primer‐Blast (https://www.ncbi.nlm.nih.gov/tools/primer‐blast). The sequences of the primers were as follows: CB‐F: 5′‐ATCAGGATTAATGGCTCCCGT‐3′; CB‐R: 5′‐AGTTGTAGCTTCTCACAGATCATT‐3′. The specificity and sensitivity of the method were evaluated on pure genomic DNA of *C. braakii* and a DNA mixture from other common bacteria in the gastric mucosa (*Lactobacillus salivarius*, *Lactobacillus mucosae*, *Streptococcus salivarius*, *Streptococcus thermophiles*, *Planomicrobium okeanokoites*, *Neisseria mucosa*, *Neisseria subflava*, *Serratia marcescens*, *Escherichia coli* and *H. pylori*). To amplify *C. braakii* from gastric samples, a total of 40 ng of tissue DNA was added to each PCR at a 62 °C annealing temperature with 35 cycles of amplification. Amplicons from 10 PCRs were directly sequenced to confirm correct amplification.

### Isolation and phenotype analyses of *C. braakii* P1

The biopsy samples were ground with sterile grinding pestles. Subsequently, the ground tissue mixture was streaked on Luria–Bertani (LB) agar plates [27] and incubated for 24 h at 37 °C. Bacterial identification was based on the results of the colony morphology and Gram staining. Further identification was conducted by amplifying bacterial 16S rRNA and sequencing. The urease activity of strain P1 was detected using the method described in the Urease Activity Detection Kit (Beijing Solarbio Science & Technology Co., Ltd., Beijing, China; Cat#BC4115). *Helicobacter pylori* 26,695 was used as a positive control. In brief, *H. pylori* 26695 or *C. braakii* P1 were quantified 5 × 10^6^ CFU·mL^−1^ following McFarland Nephelometer standards. Then, the bacterial urease hydrolyzed urea to produce ammonia‐nitrogen (NH_3_‐N) and carbonic acid. The NH_3_ was measured using the indophenol‐blue method by measuring the absorbance at 630 nm and was expressed as U/10^6^ cell (an enzyme activity unit was defined as 10^6^ cells producing 1 μg NH_3_‐N per minute). This experiment was independently replicated for three times.

To determine the survival rate of *C. braakii* in acidic conditions, strain P1 was placed in LB broth adjusted to different pH values (pH 1.0–7.0) at a final amount of 10^6^ bacterial cells and incubated at 37 °C for 2 h. The cultures were serially diluted and plated in triplicate onto LB agar plates and colonies were counted. The bacterial survival percentage was calculated [28].

### Genomic analyses of *C. braakii* P1

The *C. braakii* genome was sequenced on the PacBio (Pacific Biosciences, Menlo Park, CA, USA) platform by the Allwegene Company (Beijing, China). Raw data of the genomic sequencing was deposited in GenBank under a Bioproject number PRJNA808801. Genome annotation was carried out using dfast [29]. macsyfinder [30] was used to predict the secretion system of *C. braakii* P1 and 106 other genomes of *C. braakii* downloaded from the NCBI database. Analysis of genomic islands was performed using islandviewer 4 [31]. Virulence‐related genes were identified using the Virulence Factors of Pathogenic Bacteria Database [32].

### Cell adhesion assays

Human gastric epithelial cells AGS (BNCC338141) were purchased from Beijing BeNa Culture Collection Institute for Biological Research (Beijing, China) and human epidermoid laryngocarcinoma cells HEp‐2 (CL‐0101) were purchased from Procell Biotech (Wuhan, China).

The cells were cultured in RPMI 1640 (Procell; Cat#PM150110) supplemented with 10% fetal bovine serum (Biological Industries, Beit HaEmek, Israel; Cat#04‐001‐1ACS) in a 5% CO_2_, water‐saturated atmosphere. Assays of the adhesion of *C. braakii* to AGS or HEp‐2 cells were performed according to the method described by Li *et al*. [13]. In brief, epithelial cells were seeded onto glass coverslips in 24‐well plates at a density of 1 × 10^5^ cells per well. About 24 h later, the medium was replaced before infection with medium without fetal bovine serum. An alternative well was used for cell counting and then *C. braakii* P1 was added at a multiplicity of infection (MOI) of 100 : 1 to AGS or HEp‐2 monolayers and incubated for 2 h at 37 °C in 5% CO_2_. Each well was then rinsed five times with phosphate‐buffered saline (PBS). Cells were fixed and stained with 10% Giemsa staining solution. At least 10 pictures were taken from different fields, and the cells and adhesive bacteria were counted. The adhesion index was calculated as the number of bacteria adhered per cell [13]. The coverslips were examined under a light transmission microscope. Coculture of *C. braakii* P1 with AGS or HEp‐2 cells was repeated three times in duplicate. To determine the adhesion rate, cells were washed with PBS and lysed with RPMI 1640 containing 1% Triton X‐100 for 5 min. Serial dilutions of the lysed cells were then spread on LB agar plates. The number of adhered bacteria was determined by counting colonies on agar plates. The adhesion rate was calculated as the number of adhered bacteria divided by the number of inoculated bacteria per well ×100%.

The adhesion of strain P1 to AGS cells was further examined using transmission electron microscopy (TEM). The AGS cells cocultured with *C. braakii* P1 were washed twice, fixed overnight in 2.5% glutaraldehyde medium at 4 °C, and examined using a TEM (JEM‐1200EX; JEOL USA Inc., Peabody, MA, USA).

### Cell cytotoxicity assays

The cell cytotoxicity effect was assayed by measuring lactate dehydrogenase (LDH) release. After infection with *C. braakii* P1, the plate was centrifuged at 600 ***g*** for 5 min. LDH release from AGS or HEp‐2 cells was measured using an LDH cytotoxicity assay kit (Beyotime Biotechnology, Shanghai, China; Cat#C0016) according to the manufacturer's instructions. The absorbance at a wavelength of 490 nm was determined. The LDH release percentage was then calculated [33]. All tests were repeated three times in duplicate.

### RNA extraction and quantitative real‐time PCR (qPCR)

Total cellular RNA was extracted by using a total RNA extraction kit (Tiangen, China; Cat#DP419). Reverse transcription of the total RNA was performed using FastKing gDNA Dispelling RT SuperMix (Tiangen, Beijing, China; Cat#KR118). The mRNA levels of *IL‐8* and *TNF‐α* were quantified subsequently using qPCR as previously reported [34, 35].

### ELISA detection of *IL‐8* and *TNF‐α*

The levels of IL‐8 and TNF‐α secreted into the cell culture medium after infection with strain P1 were determined using human cytokine ELISA kits (Elabscience Biotechnology, Wuhan, China; Cat#E‐EL‐H6008 and Cat#E‐EL‐P0010c) according to the manufacturer's instructions. The absorbance at a wavelength of 450 nm was determined. Cytokine concentrations were then calculated from a standard curve.

### Statistical analyses

All analyses were performed using graphpad prism 7.0 (Graphpad Prism Software Inc., San Diego, CA, USA) and spss version 23 (IBM SPSS, Chicago, IL, USA) software. The data are presented as the mean ± SD. Differences between mean values were compared using the unpaired two‐tailed Student's *t*‐test or two‐way analysis of variance (ANOVA). Multiple linear regression analysis was used to assess the association between bacterial infection and the activity score and inflammatory score of the gastric mucosa. *P* < 0.05 was considered statistically significant.

## Results

### Association between *C. braakii* and gastric mucosal inflammation

The status of *C. braakii* was determined in 172 patients with CG. Of them, 61 (35.5%) patients were positive for *C. braakii*. The prevalence of *C. braakii* in *H. pylori‐*negative patients (49.1%, 56/114) was much higher than that in *H. pylori‐*positive patients (8.6%, 5/58). The demographic data of patients with CG were summarized in Table 1. The correct identification of *C. braakii* was validated by sequencing 10 randomly selected PCR products. Multiple linear regression analyses showed that both *H. pylori* and *C. braakii* infection were positively correlated with the inflammatory score of CG. The regression coefficients were 1.315 and 0.352, suggesting an association between the presence of *C. braakii* and enhanced inflammatory severity. However, *C. braakii* infection showed no correlation with the activity score.

**Table 1:** Demographic data of patients with chronic gastritis.

Characteristics	Number
Patients	172
Gender	
Male; *n* (%)	91 (52.9)
Female; *n* (%)	81 (47.1)
Age; mean (min–max)	50.4 years (15–86)
Gastritis inflammatory score	
1; *n* (%)	67 (39.0)
2; *n* (%)	56 (32.6)
3; *n* (%)	49 (28.4)
Gastritis activity score	
0; *n* (%)	70 (40.7)
1; *n* (%)	66 (38.4)
2; *n* (%)	31 (18.0)
3; *n* (%)	5 (2.9)
Intestinal metaplasia	
Yes; *n* (%)	77 (44.8)
No; *n* (%)	95 (55.2)
HP infection	
Negative; *n* (%)	114 (66.3)
Positive; *n* (%)	58 (33.7)
*Citrobacter braakii* infection	
Negative; *n* (%)	111 (64.5)
Positive; *n* (%)	61 (35.5)

### Genomic characterization of *C. braakii* P1 from the stomach

**Fig. 1:**
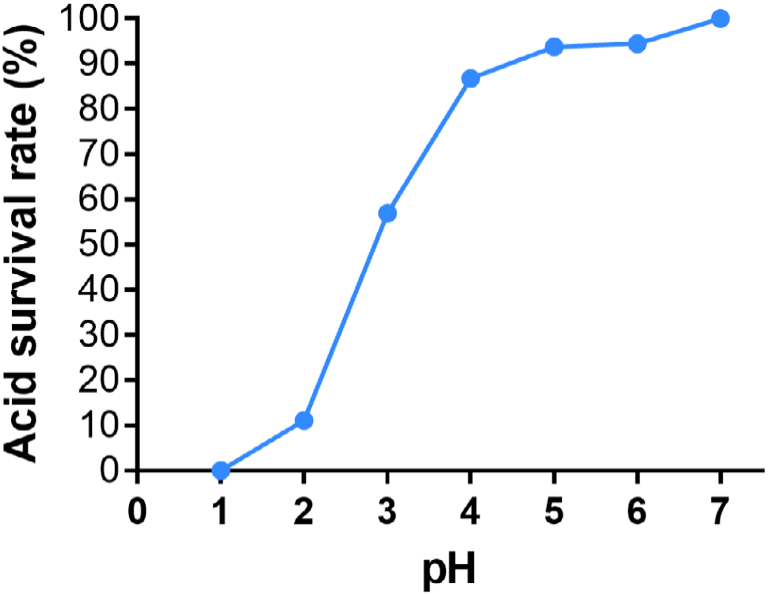
Survival rate of *Citrobacter braakii* P1 under acidic conditions. The survival rate of *C. braakii* P1 was determined after incubation in medium with different pH values (pH 1.0–7.0) for 2 h.

To further explore the pathogenesis of *C. braakii* in the gastric mucosa, *C. braakii* P1 was isolated from the antrum of an *H. pylori*‐negative patient. The urease activity of the isolate was 0.014 ± 0.001 U/10^6^ cell, which was lower than that of *H. pylori* 26695 (0.022 ± 0.002 U/10^6^ cell). *Citrobacter braakii* P1 showed strong acid tolerance. At pH 4–7, its survival rate was close to 100%, and even at pH 2, the survival rate of strain P1 remained 11.1% (Fig. 1).

*Citrobacter braakii* strain P1 was sequenced and annotated. The genome was 5,081,794 bp long, with a GC content of 52.1%. It contained 4803 coding sequences (CDSs), 25 rRNAs, and 87 tRNA genes. Strain P1 possessed *ureA*, *ureB*, and *ureC* genes encoding three subunits of urease. The amino acid sequence of urease from strain P1 shared 59.6% identity with *H. pylori* urease.

#### Potential secretion systems

**Fig. 2:**
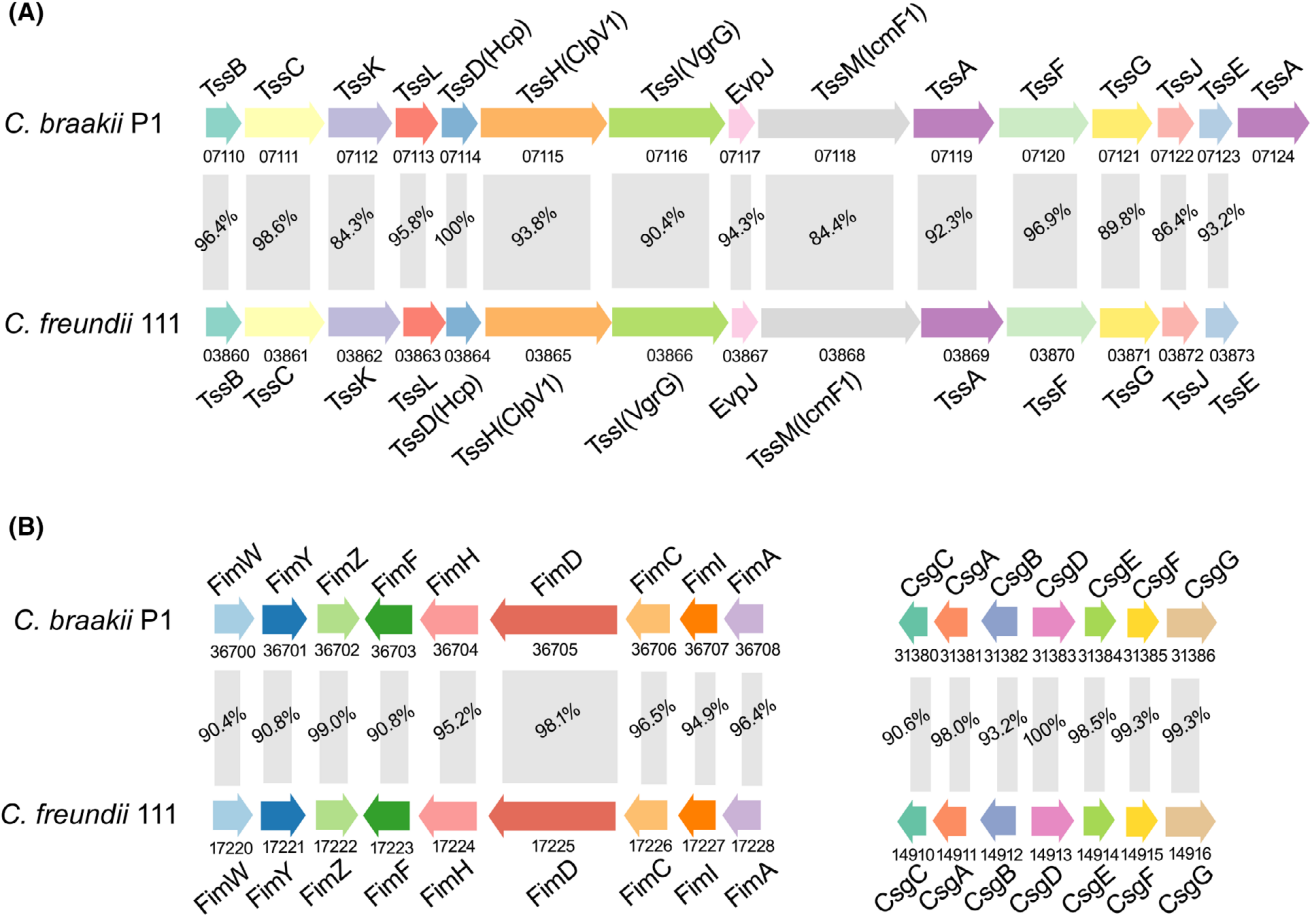
Predicted T6SS and adhesion‐related *fim* and *csg* gene clusters in *Citrobacter braakii* P1. (A) Genetic organization of the T6SS gene cluster and (B) adhesion‐related *fim* gene cluster and *csg* curli fimbriae gene cluster in strain P1 compared with those of *C. freundii* strain 111 (PRJNA592134). Genes are represented by arrows indicating the direction of translation. Protein names encoded by genes are shown above, and locus tags are shown below. Conserved T6SS components between the two species are highlighted in the same color. The similarity of the amino acid sequence between the two strains is shown in between.

*Citrobacter braakii* strain P1 possessed a complete type I secretion system (T1SS), T5aSS and T6SS and a nearly complete T2SS, lacking only GspN, which was not present in every species with a functional T2SS system and is not required for secretion in *Acinetobacter baumannii* and *Klebsiella oxytoca* [36, 37, 38]. The T6SS cluster in strain P1 contained 13 genes, including the effector proteins Hcp and VgrG encoded by *tssD* and *tssI* (Fig. 2A). Prediction of the secretion systems in a total of 106 *C. braakii* genomes available in GenBank demonstrated that none of them possessed a complete T6SS. A total of 14.2% (15/106) of the *C. braakii* genomes lacked only one gene, and 9.4% (10/106) lacked two genes. Interestingly, analysis of genomic islands revealed that the middle part of the T6SS was a potential pathogenicity island that was possibly acquired horizontally. This segment consisted of five genes, including *tssI*, encoding the effector protein VgrG. The amino sequence of T6SS genes showed high similarity to that of the T6SS in *Citrobacter freundii* (*C. freundii*), strongly suggesting the similar functions of the T6SS between the two species (Fig. 2A).

#### Predicted adhesins

Twenty‐six of the 86 virulence genes identified in *C. braakii* P1 were possibly involved in bacterial adhesion. The genome of strain P1 had a complete type I pilus *fim* gene cluster consisting of *fimAICDHF* and the regulatory genes *fimZ*, *fimY*, and *fimW*, and a *csg* gene cluster including the *csgBAC* and *csgDEFG* operons, which encoded the curli fimbriae or thin aggregative fimbriae (Fig. 2B). Gene clusters with highly similar amino sequences were found in *C. freundii* with an identity of 90.4–100%. In addition, the potential adhesins MisL, EcpA, and PilW were also predicted in strain P1.

### Adherence of *C. braakii* P1 to gastric epithelial cells

**Fig. 3:**
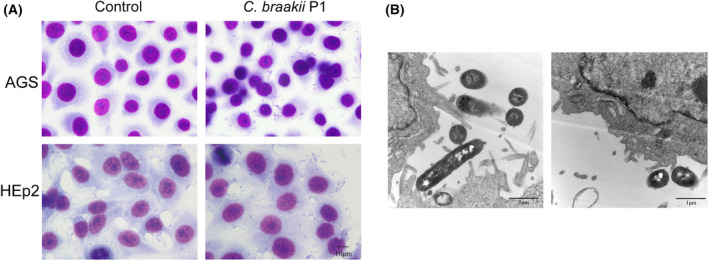
Adhesion of *Citrobacter braakii* P1 to AGS cells. (A) Representative images of Giemsa‐stained AGS and HEp‐2 cells infected with *C. braakii* P1 at an MOI of 100 for 2 h. Scale bar, 10 μm. (B) Transmission electron micrographs showing the adherence of *C. braakii* strain P1 to AGS cells. Scale bar, 1 μm.

To assess the affinity of *C. braakii* to gastric epithelial cells, strain P1 was used to infect AGS cells at an MOI of 1 : 100. Microscopy examinations of the coverslips demonstrated that strain P1 attached to the surface of AGS epithelial cells in a diffuse manner (Fig. 3A). Adherence of strain P1 to AGS cells was confirmed by TEM examinations. Strain P1 exhibited close contact with microvilli and the surface of AGS cells (Fig. 3B). In contrast, the adherence to nongastric HEp‐2 cells by strain P1 showed a scattered pattern. Consistent with this, the adhesion rate of strain P1 to AGS was 25.9%, which was much higher than that of HEp‐2 cells (10.7%, *P* < 0.001). The adhesion index of *C. braakii* strain P1 to AGS was also higher than HEp‐2 cells (8.34 ± 0.68 vs. 2.46 ± 0.38), which supported the results above.

### Cell cytotoxicity of *C. braakii* P1

**Fig. 4:**
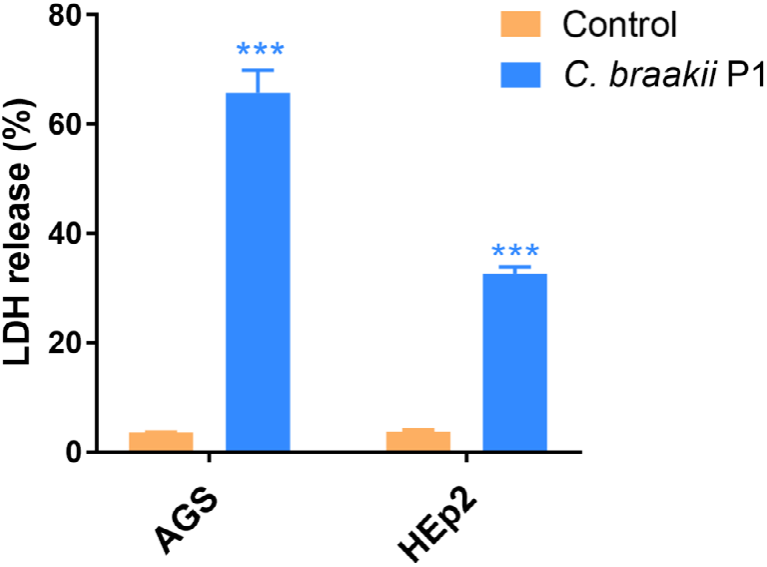
Cell cytotoxicity of *Citrobacter braakii* P1. The cell cytotoxicity effect of *C. braakii* P1 was examined by measuring LDH release from AGS and HEp‐2 cells. Data are shown as means ± SD based on three independent experiments. ***P < 0.001 by unpaired two‐tailed Student's *t*‐tests.

To determine the cytotoxicity of *C. braakii* to gastric epithelial cells, the amount of LDH released into the supernatant of AGS cells cocultured with strain P1 was measured. The LDH release percentage was significantly higher in AGS cells (65.8%, Fig. 4) than in uninfected cells (3.5%, *P* < 0.001). This suggested a high cytotoxicity of *C. braakii* to gastric epithelial cells. However, HEp‐2 cells cocultured with strain P1 showed a moderate increase in the LDH release percentage (32.6%).

### Increased expression of IL‐8 and TNF‐α in gastric epithelial cells

**Fig. 5:**
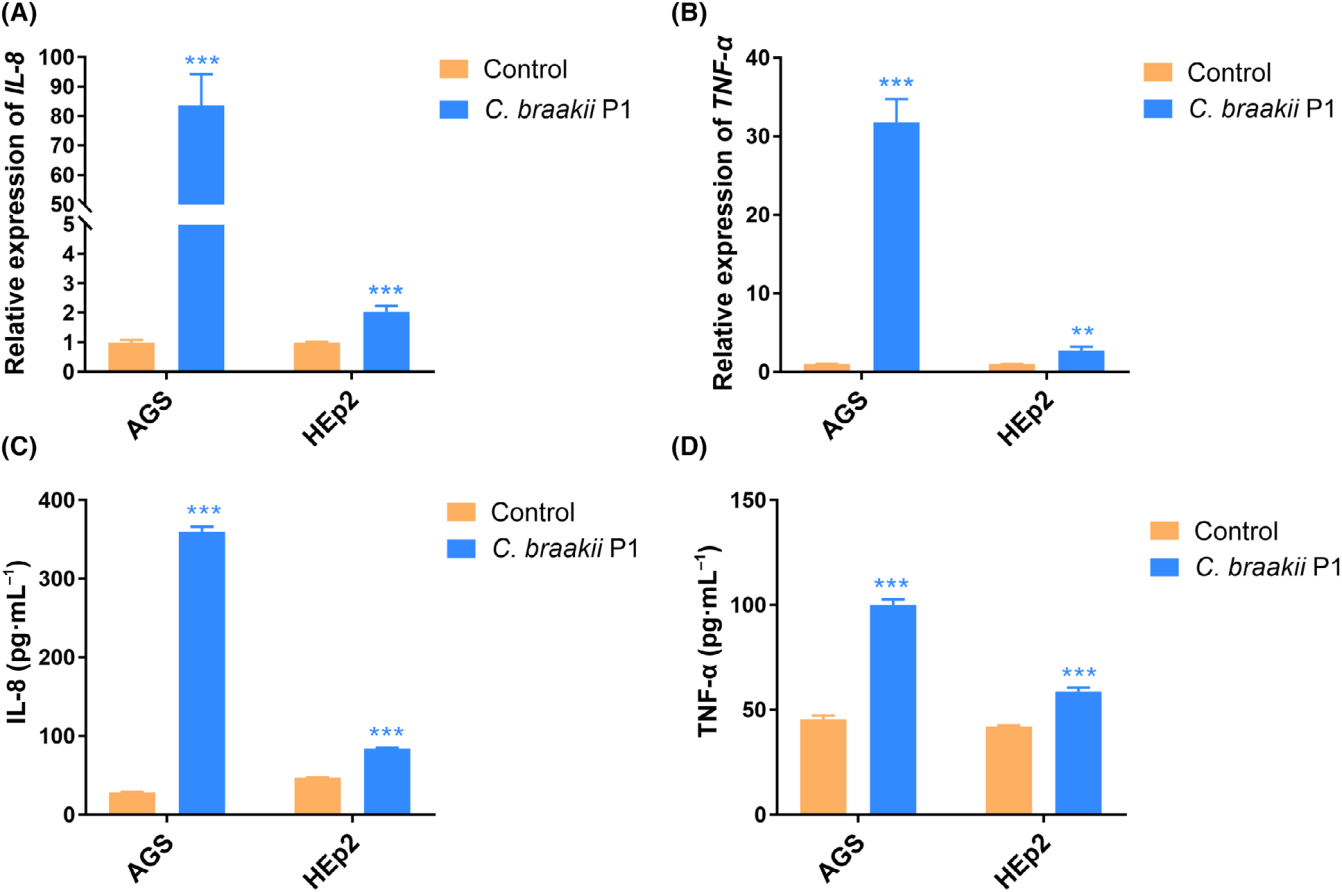
Inflammatory response induced by *Citrobacter braakii* P1 from epithelial cells. (A) mRNA level of *IL‐8* in AGS and HEp‐2 cells induced by *C. braakii* P1 infection. (B) mRNA level of *TNF‐α*. (C) Amount of IL‐8 secreted into cell culture medium induced by *C. braakii* P1 infection. (D) Amount of TNF‐α secreted into cell culture medium. Data are shown as means ± SD based on three independent experiments. **P < 0.01; ***P < 0.001 by unpaired two‐tailed Student's *t*‐tests.

The mRNA levels of *IL‐8* and *TNF‐α* were determined in AGS cells after coculture with *C. braakii* strain P1 for 24 h. The mRNA expression levels of both genes showed a very significant increase compared with those in uninfected AGS cells (Fig. 5A,B). The expression level increased approximately 83‐ and 32‐fold for *IL‐8* and *TNF‐α*, respectively. Consistent with this finding, the amounts of IL‐8 and TNF‐α proteins released into the culture medium were significantly increased in AGS cells cocultured with P1 compared with uninfected AGS cells (Fig. 5C,D). Coculture of HEp‐2 cells with *C. braakii* strain P1 also resulted in a significant increase in the mRNA or protein expression levels of both *IL‐8* and *TNF‐α* (Fig. 5). However, the increase in the expression appeared to be moderate. The mRNA level increased approximately twofold increase for both *IL‐8* and *TNF‐α*, while the protein expression level increased 1.8‐ and 1.4‐fold for *IL‐8* and *TNF‐α*, respectively (Fig. 5).

## Discussion

The contributions of *C. braakii* to the occurrence of gastric mucosal inflammation remain to be clarified. Findings from this study demonstrated that the presence of *C. braakii* was associated with the severity of gastric mucosal inflammation. The urease‐positive, cytotoxic *C. braakii* was capable of adhering to gastric epithelial cells and inducing the release of the proinflammatory cytokines IL‐8 and TNF‐α, which contribute to the development of mucosal inflammation.

*Citrobacter braakii* has been isolated from the intestinal tract and extraintestinal tissues [21, 22]. It occasionally causes a range of infections [39, 40]. As members of the microbiome, *Citrobacter* species can persist in the stomach [16]. In this study, we designed specific PCR primers to detect the presence of *C. braakii* in antral mucosal biopsy samples from patients with CG. Our results found that *C. braakii* was prevalent in patients with CG, with a higher prevalence in *H. pylori‐*negative patients. This is consistent with findings from molecular analyses of the gastric microbiome showing that *C. braakii* is dominant in the gastric antrum of *H. pylori‐*negative patients with CG. Our results demonstrated that both *C. braakii* and *H. pylori* were associated with the degree of mucosal inflammation. These findings demonstrated a likely contribution of *C. braakii* to the occurrence of chronic mucosal inflammation in the stomach.

Our results showed that strain P1 isolated from the stomach of an *H. pylori‐*negative patient with CG possessed urease activity and a strong capacity for acid resistance. Its genome contains the *ureA*, *B*, and *C* genes, encoding the subunits of urease. These results suggested that *C. braakii* is capable of surviving in acidic conditions, which explains its persistence in the human stomach [41, 42].

Certain *C. braakii* strains from environmental or human sources are capable of adhering to HEp‐2 cells [25], which are originated from a human laryngeal carcinoma and usually applied to experiments of bacterial adhesion ability and cytotoxicity in previous study [21, 22]. Meanwhile, human pathogens have adapted to thrive within the host specific cell environment [43]. In this study, *C. braakii* P1 was isolated from stomach. To explore whether *C. braakii* could attach to gastric epithelial cells, we further analyzed the adherence of strain P1 to AGS cells. Our results demonstrated that *C. braakii* P1 attached to AGS cells with a diffuse pattern. Furthermore, its affinity to AGS cells was much higher than that to HEp‐2 cells. These findings suggest that *C. braakii* from the stomach had a relative high capacity to adhere to gastric epithelial cells. The high affinity of *C. braakii* P1 to gastric epithelial cells may facilitate its persistence in the stomach and promote its interaction with the gastric epithelium. Genomic analyses of strain P1 identified a number of potential adhesins, including gene clusters encoding type 1 fimbriae and curli fimbriae. Their involvement in attachment to gastric epithelial cells requires further elucidation.

In this study, we found that *C. braakii* P1 from the stomach had higher cytotoxicity to AGS than to HEp‐2 cells. Cytotoxic effects on epithelial cells have been found in some *C. braakii* strains isolated from foods, the intestine and the urinary tract [21, 22, 25]. Our results further demonstrated that AGS cells infected with *C. braakii* P1 showed markedly increased mRNA and protein expression of *IL‐8* and *TNF‐α*. The fold increase in *IL‐8* and *TNF‐α* expression induced by *C. braakii* P1 in AGS cells was much higher than that in HEp‐2 cells. These findings suggest that *C. braakii* recruits inflammatory cells by inducing the expression of IL‐8 and TNF‐α inflammatory cytokines, thus initiating mucosal inflammation and contributing to the development of gastritis [44, 45, 46].

Genomic analyses of *C. braakii* P1 revealed the presence of a complete T1SS, T2SS, T5aSS, and T6SS. These secretion systems are likely involved in adhesion, cytotoxicity, and induction of inflammatory responses [47]. A complete T6SS was found only in strain P1, in contrast to the presence of a nearly complete T6SS in 106 *C. braakii* genomes available in GenBank. Furthermore, *tssI* encoding the effector protein VgrG in the T6SS of strain P1 was possibly acquired horizontally, indicating potential changes in VgrG‐related functions. The bacterial T6SS possesses a vast effector repertoire targeting the cell wall, cell membrane, and nucleic acids of neighboring bacteria [48]. The delivery of T6SS effectors enhances bacterial competition with other bacteria in the microbiome [48, 49] and facilitates bacterial colonization of the host [50, 51]. Thus, *C. braakii* likely has a selective advantage in overgrowth in the human stomach. Moreover, the *C. braakii* T6SS is possibly involved in cytotoxicity to AGS cells [52, 53, 54]. The VgrG, Hcp‐2 and ClpV proteins of the T6SS participate in the cytotoxicity of *C. freundii* to epithelial cells [54]. In addition, the *C. braakii* T6SS is potentially involved in the induction of the inflammatory response of gastric epithelial cells [55, 56]. Our study still has several limitations. At present, we have predicted a complete secretion system of T6SS in *C. braakii* P1; however, it needs experimental evidence. Additionally, cytotoxicity and proinflammatory response induced by *C. braakii* have confirmed in gastric epithelial cell, while the experiments of Germ‐free mice *in vivo* need to be explored for further validation.

In summary, our study demonstrated that *C. braakii* was prevalent in the stomach and was associated with the severity of gastric mucosal inflammation. It was capable of adhering to gastric epithelial cells and inducing the release of the proinflammatory cytokines IL‐8 and TNF‐α, thus contributing to the development of gastric mucosal inflammation. Additionally, *C. braakii* P1 encoded urease activity, acid resistance capacity and a potential antibacterial T6SS in its genome, which could facilitate its colonization and persistence in the human stomach. The pathogenicity of *C. braakii*, as a member of the gastric microbiome, is likely involved in CG, leading to the progression to more severe pathologies of the gastric mucosa.

## Conflict of interest

The authors declare no conflict of interest.

## Author contributions

MY, FX, and CX obtained the data; TY, YW, and SL provided technical support; QD and LW conceived and wrote this manuscript.

## Data Availability

The genomic sequencing raw data that support the findings in this study are openly available in the Genbank at NCBI at https://www.ncbi.nlm.nih.gov/bioproject/?term=PRJNA808801, accession number [PRJNA808801]. The data that support the findings of this study are available from the corresponding author [wanglili1@qdu.edu.cn] upon reasonable request.
